# Supplementary material for: Neutrophils induce paracrine telomere dysfunction and senescence in ROS‐dependent manner
Source: EMBO J. 2021 Mar 25;40(9):e106048. doi: 10.15252/embj.2020106048 (PMC8090854; doi:10.15252/embj.2020106048)
Supplement: Supplementary file 1 — Appendix [file EMBJ-40-e106048-s003.pdf]

Lagnado *et al*. Appendix

Table of contents:

2 Appendix Supplementary Figures

1 Appendix Supplementary Table

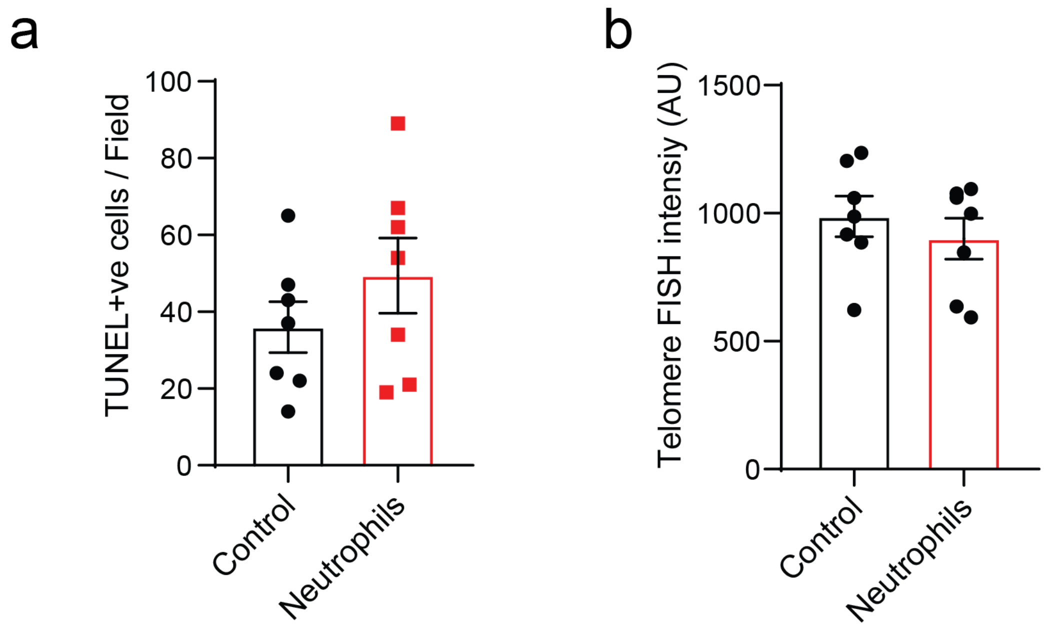

Appendix Figure S1. Neutrophils do not induce cell-death or telomere shortening in human precision cut liver slices (PCLS). a) Number of TUNEL stained positive cells per field; **b)** Mean telomere FISH intensity. Data are from n=7 patients.

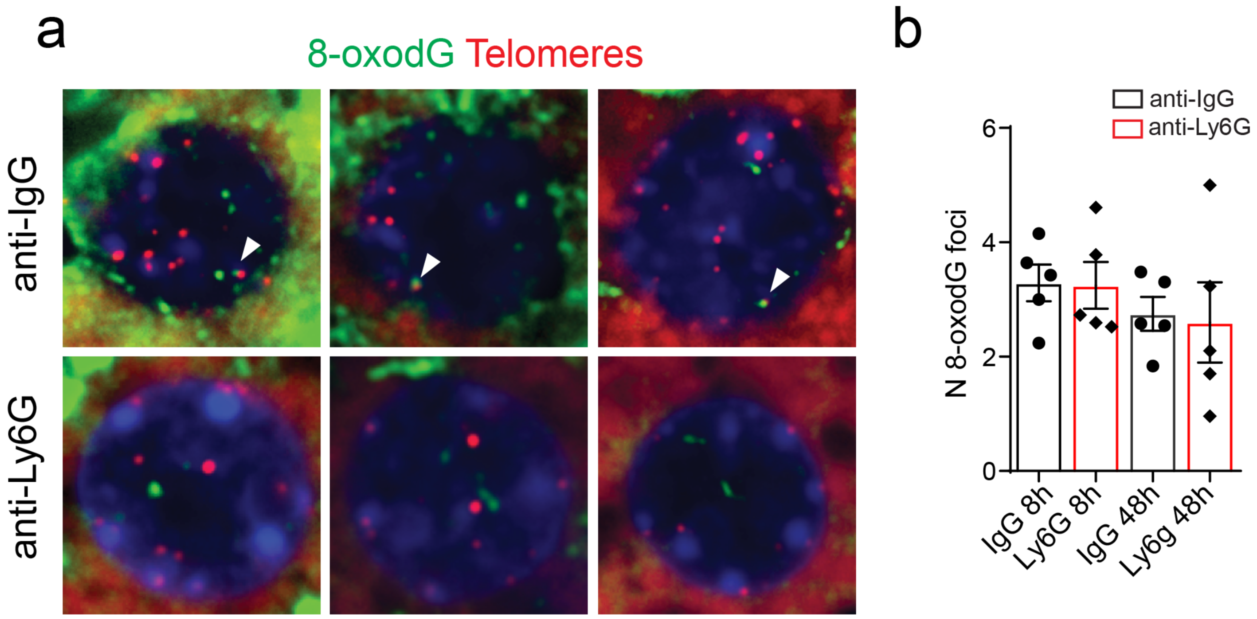

Appendix Figure S2. Neutrophil depletion reduces telomere-associated 8-oxodG foci, but not total 8-oxodG foci a) Representative immuno-FISH micrographs using antibody against 8-oxodG and Cy-3-labelled telomere-specific (CCCTAA) peptide nucleic acid probe in hepatocytes 8 and 48 hours after injection of CCl_4 pre-treated with neutrophil neutralizing antibody against Ly6G (or with IgG control); **b)** graph shows quantification of total number of nuclear 8-oxodG foci.

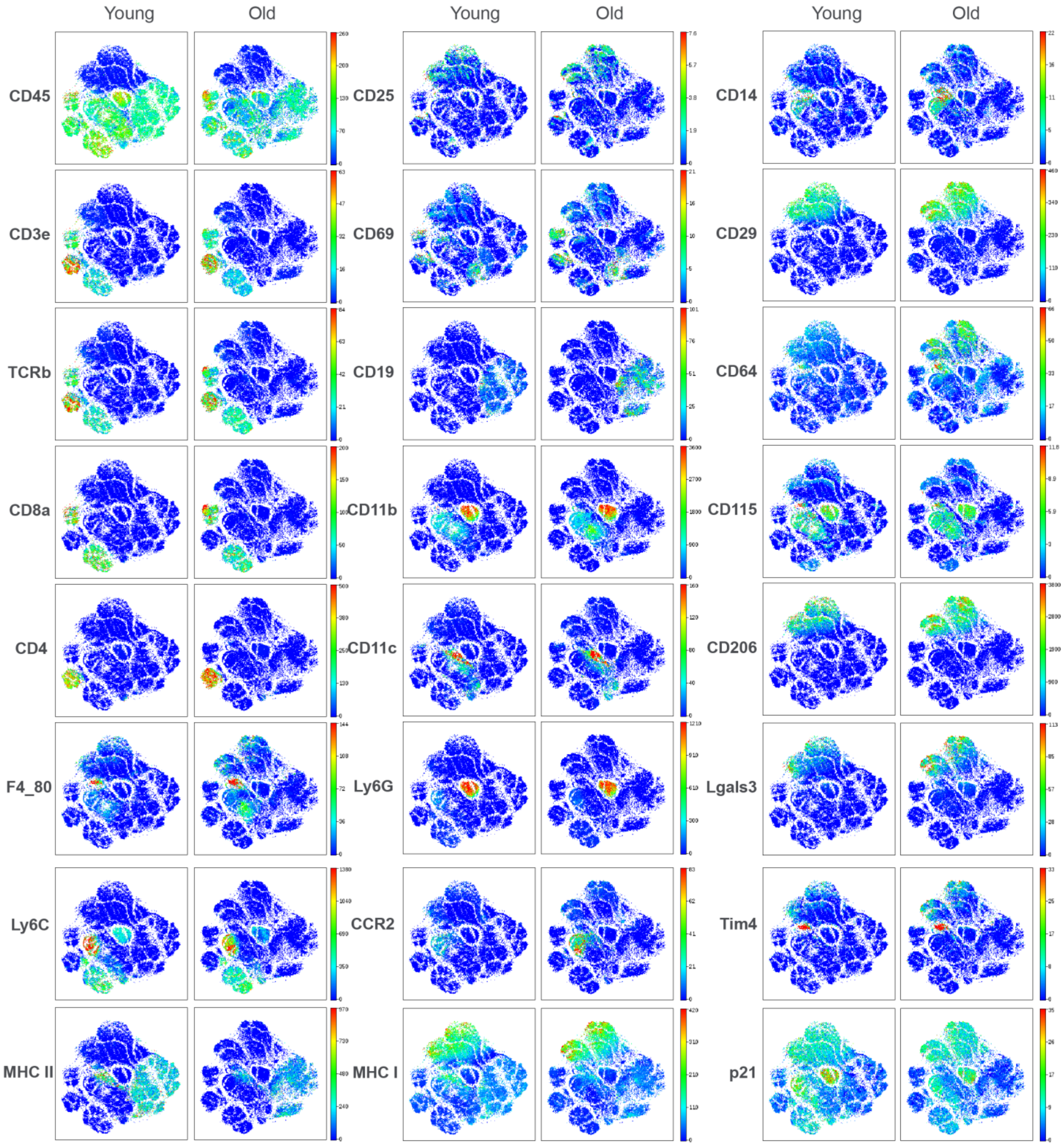

Appendix Figure S3- Intrahepatic leukocyte profiling by mass CyTOF. Representative tSNE plots for each marker used. Red indicates high frequency categorization of cells to a cluster; blue indicates low frequency; n = 5 per age group.

Species	Label	Target	Clone	Company	Cat. #
Ms	089Y	CD45	30-F11	Fluidigm	3089005B
Ms	141Pr	Lgals3	202213	R&D Systems	MAB1197
Ms	142Nd	CD11c	N418	Fluidigm	3142003B
Ms	143Nd	TCRb	H57-597	Fluidigm	3143010B
Ms	144Nd	MHC Class I	28-14-8	Fluidigm	3144016B
Ms	145Nd	CD69	H1.2F3	Fluidigm	3144016B
Ms	149Sm	Tim4	RMT4-54	BioLegend	130002
Ms	151Eu	CD25 (IL-2R)	3C7	Fluidigm	3150002B
Ms	152Sm	CD3e	145-2C11	Fluidigm	3152004B
Ms	153Eu	CD29 / Itgb1	9EG7	BD Biosciences	550531
Ms	156Gd	CCR2	475301	R&D Systems	MAB55381
Ms	159Tb	F4/80	BM8	Fluidigm	3159009B
Ms	160Gd	CD64	290322	R&D Systems	MAB20741
Ms	161Dy	Ly6G	1A8	Biolegend	127637
Ms	163Dy	CD4	RM4-5	Biolegend	100561
Ms	165Ho	CD14	Sa14-2	Biolegend	123321
Ms	166Er	CD19	6D5	Fluidigm	3166015B
Ms	168Er	CD8a	53-6.7	Fluidigm	3168003B
Ms	169Tm	CD206 (MMR)	C068C2	Fluidigm	3169021B
Ms	171Yb	CD11b (Mac-1)	M1/70	Biolegend	101249
Ms	174Yb	CD115/CSF1R	AFS98	Biolegend	135521
Ms	175Lu	Ly6C	HK1.4	Biolegend	128039
Ms	209Bi	I-A/I-E	M5/114.15.2	Fluidigm	3209006B
Ms	155Gd	p16	EPR20418	Abcam	ab232402
Ms	176Yb	p21	F-5	Santa Cruz	sc-6246

Appendix Table S1: Table of antibodies used for CyTOF.
