# Supplementary material for: Neutrophils induce paracrine telomere dysfunction and senescence in ROS‐dependent manner
Source: EMBO J. 2021 Mar 25;40(9):e106048. doi: 10.15252/embj.2020106048 (PMC8090854; doi:10.15252/embj.2020106048)
Supplement: Supplementary file 2 — Expanded View Figures PDF [file EMBJ-40-e106048-s004.pdf]

Expanded View Figures

Figure EV1. Neutrophils induce paracrine senescence.

A Age of neutrophil healthy donors used in independent co-culture experiments. Data are mean ± SEM of $n = 3$.
B Table summarizing gender and donor age as well as % of neutrophil purity.
C Growth curves comparing population doublings in controls and MRC5 co-cultured with non-primed neutrophils. Blue arrow indicates time point in which neutrophils were added to culture for consecutive 3 days.
D Superoxide anion levels in neutrophils following treatment with different concentrations of LPS. The highest increase was observed after 1 h treatment with 100 ng/ml. Data are mean ± SEM of $n = 5$ independent experiments.
E Representative Western blot of p15 and tubulin expression 8 and 28 days after neutrophil co-culture.
F Quantification of p15 expression relative to tubulin. Data are mean ± SEM of three independent experiments.
G Expression of selected secreted factors (from cytokine array depicted in Figure 1j) at 8 and 28 days after neutrophil co-culture. Data are mean ± SEM of three independent experiments.

Data information: Statistical analysis was performed using one-way ANOVA (Holm–Sidak method) for multiple comparisons and two-tailed *t*-test for single comparisons. $^{**}P < 0.01$; $^{*}P < 0.04$.

© 2021 The Authors

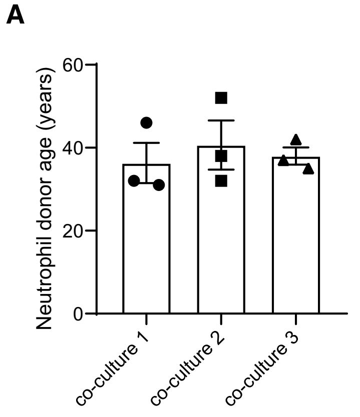

B

Gender	Donor age	neutrophil purity (%)
f	32	97.4
f	46	96.8
f	31	97
f	38	98.5
f	42	98.1
f	35	98.6
f	37	97.3
m	52	96.3
m	32	97.9

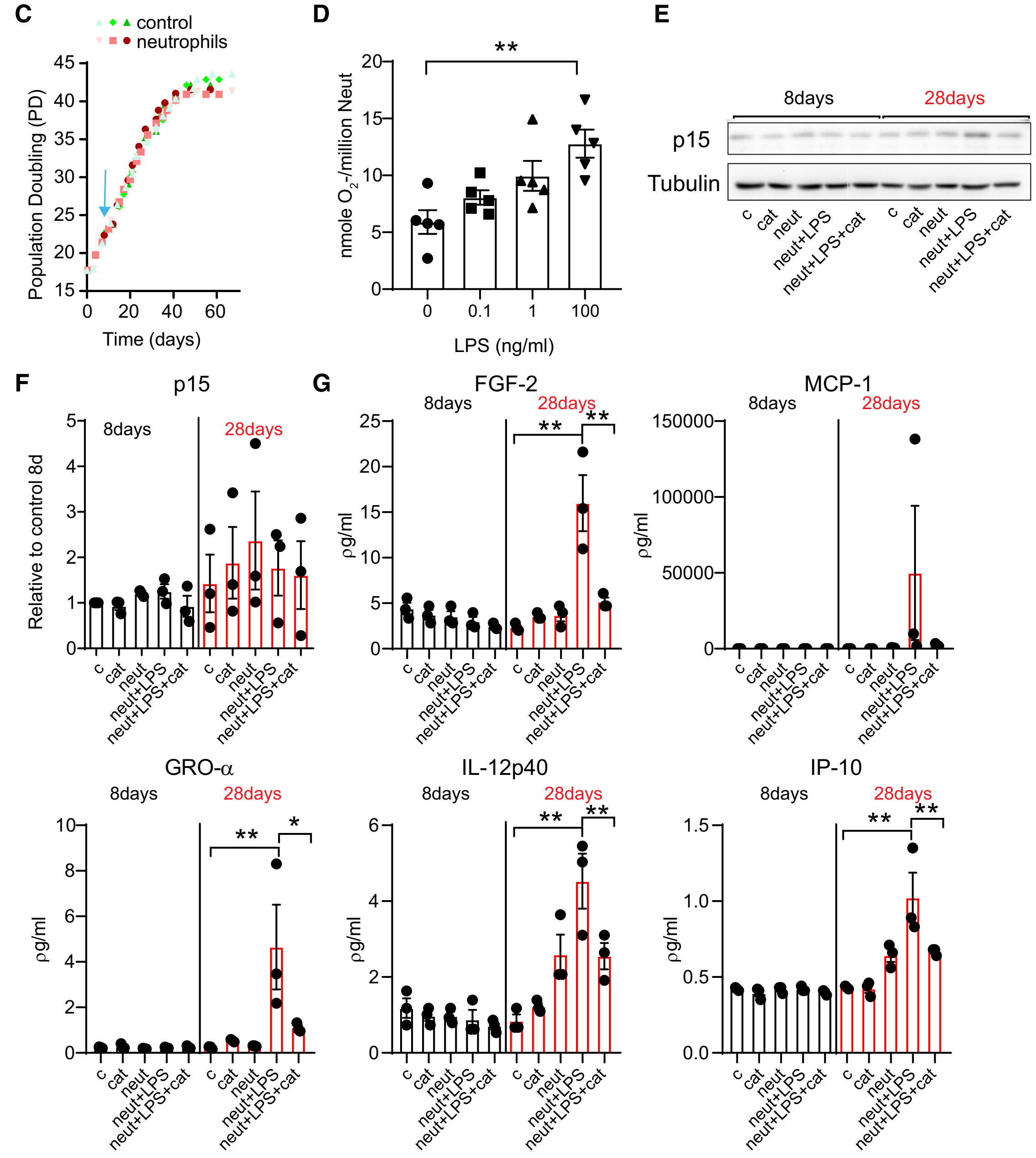

Figure EV1.

© 2021 The Authors

Figure EV2. Neutrophils induce a DNA damage response in bystander cells.

A, B Effect of neutrophil and recombinant catalase co-culture on (A) mean number of γH2A.X foci per cell and (B) % tail intensity measured by Alkaline Comet assay. Data are mean ± SEM of $n = 3$ independent experiments.

C–E Effect of hTERT expression (8 and 20 days after primed neutrophil co-culture) on (C) number of 53BP1 foci, (D) expression of $p16^{INK4A}$ and p15 and (E) $p16^{INK4A}$ mRNA levels. Data are mean ± SEM of $n = 2–3$ independent experiments.

F Representative micrograph showing nuclear localization of hTERT 4 days after co-culture with primed neutrophils.

Data information: Statistical analysis was performed using one-way ANOVA (Holm–Sidak method) for multiple comparisons and two-tailed *t*-test for single comparisons. $^{**}P < 0.01$; $^{*}P < 0.04$.

 © 2021 The Authors

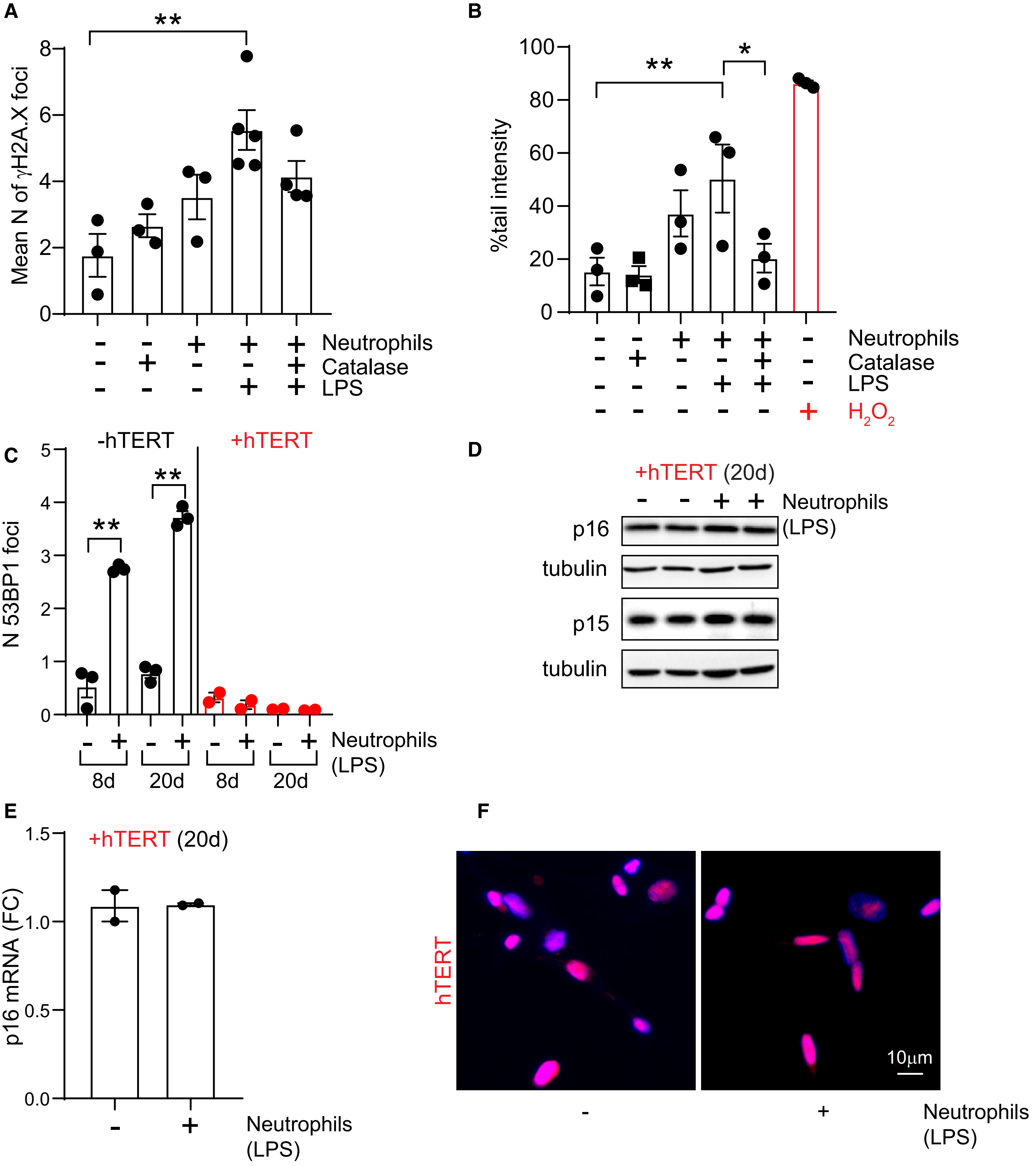
A
**
Mean N of γH2A.X foci
8
6
4
2
0
- - + + + Neutrophils
- + - - + Catalase
- - - + + LPS
B
*
**
%tail intensity
100
80
60
40
20
0
- - + + + - Neutrophils
- + - - + - Catalase
- - - + + - LPS
- - - - - + H2O2
C
-hTERT
+hTERT
**
**
N 53BP1 foci
5
4
3
2
1
0
- + - + - + - + Neutrophils
(LPS)
8d 20d 8d 20d
D
+hTERT (20d)
- - + + Neutrophils
(LPS)
p16
tubulin
p15
tubulin
E
+hTERT (20d)
p16 mRNA (FC)
1.5
1.0
0.5
0.0
- + Neutrophils
(LPS)
F
hTERT
10μm
- + Neutrophils
(LPS)

Figure EV2.

Figure EV3. Neutrophil-induced paracrine senescence requires cell-to-cell contact.

A Schematic depicting experimental design. MRC5 fibroblasts were (i) co-cultured with neutrophils; (ii) exposed to conditioned media from neutrophils; or (iii) co-cultured with neutrophils in transwell inserts for 3 days (neutrophils were primed with LPS and replenished every 24 h).

B Effect of neutrophil co-culture, conditioned media (CM) or transwell co-culture (TW) on population doublings (Data are mean ± SEM of $n = 3$ independent experiments).

C, D (C) Mean number of TAF and (D) % TAF-positive cells 8 and 20 days after neutrophil direct or indirect co-culture.

E Representative Immuno-FISH micrographs (telomere FISH and 53BP1).

F–H (F) p21 mRNA, (G) p21 protein expression and (H) p16 protein expression levels 20 days after neutrophil direct or indirect co-culture.

I Representative Western blot detecting expression of $p16^{INK4A}$, p21 and tubulin.

J, K (J) IL-6 mRNA and (K) IL-8 mRNA expression 20 days after neutrophil direct or indirect co-culture.

Data information: Data are mean ± SEM of $n = 3$. Statistical analysis was performed using one-way ANOVA (Holm–Sidak method) for multiple comparisons and two-tailed *t*-test for single comparisons. $^{**}P < 0.01$; $^{*}P < 0.04$.

 © 2021 The Authors

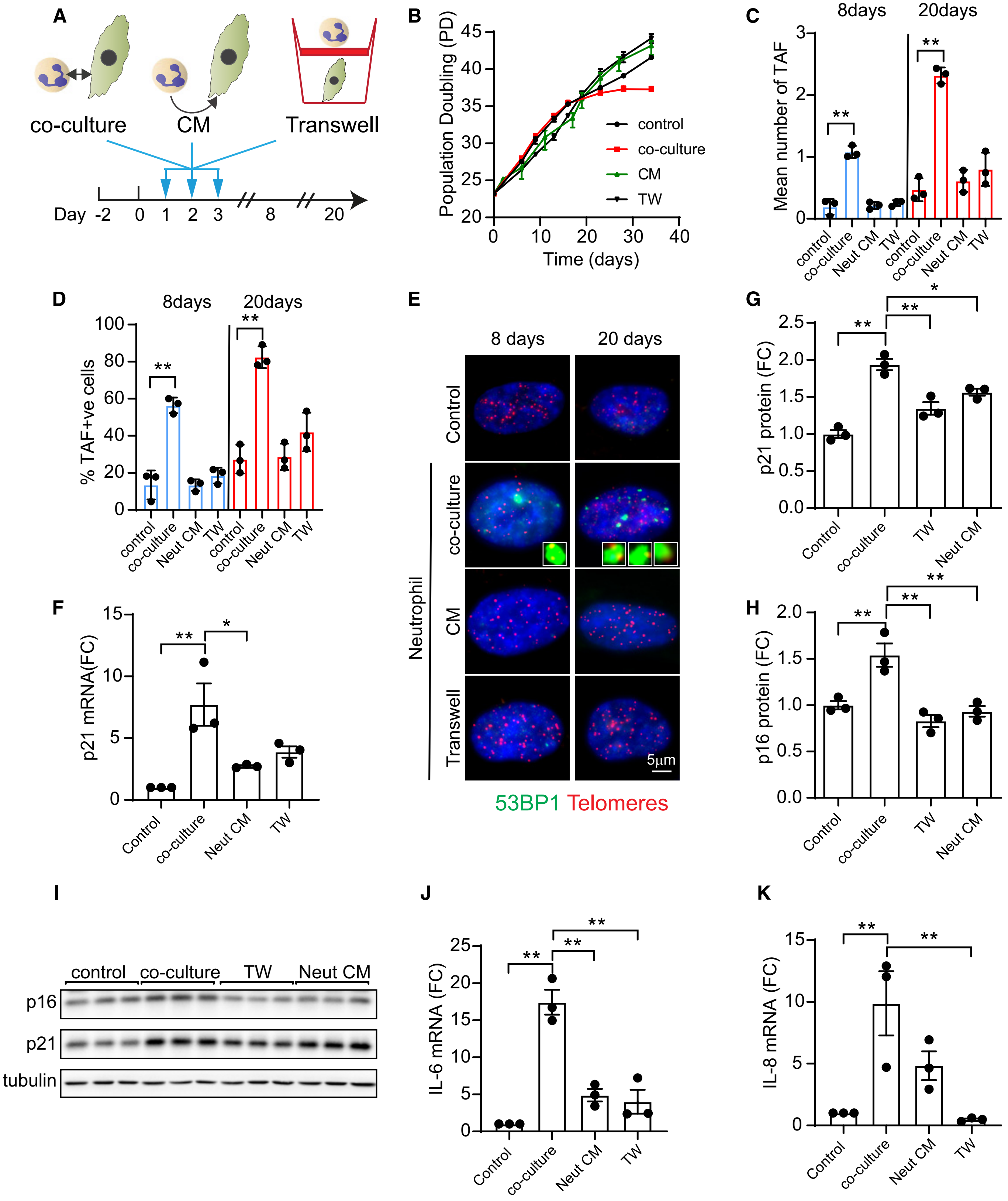
A
co-culture
CM
Transwell
Day -2 0 1 2 3 8 20
B
Population Doubling (PD)
Time (days)
control
co-culture
CM
TW
C
8days
20days
Mean number of TAF
control
co-culture
Neut CM
TW
D
% TAF+ve cells
E
8 days
20 days
Control
co-culture
CM
Transwell
Neutrophil
5μm
53BP1 Telomeres
F
p21 mRNA(FC)
G
p21 protein (FC)
H
p16 protein (FC)
I
control
co-culture
TW
Neut CM
p16
p21
tubulin
J
IL-6 mRNA (FC)
K
IL-8 mRNA (FC)

Figure EV3.

Figure EV4. Neutrophils induce telomere dysfunction and senescence-associated pathways *in vivo*.

A IL-1α mRNA expression in hepatocytes 8 and 48 h after injection of CCl_4 pre-treated with neutrophil neutralizing antibody against Ly6G (or with IgG control).
B Representative micrographs of Lamin B1 expression in hepatocytes 48 h after injection of CCl_4.
C Mean Lamin B1 intensity.
D Nuclear area comparison of p21 negative (-ve) or positive (+ve) hepatocytes 48 h after injection of CCl_4.
E Histograms representing nuclear area distributions of TAF negative (−ve) or positive (+ve) hepatocytes 48 h after injection of CCl_4. Wild-type and $Tlr2^{-/-}$ mice were injected with a single intraperitoneal injection of CCl_4 at a dose of 2 µl/g body weight and were sacrificed 24 h, 48 h and 72 h after injection.
F–I Graphs show quantification of (F) γH2A.X, (G) mean number of Telomere-associated foci (TAF), (H) % of TAF-positive hepatocytes (in PCNA negative hepatocytes) and (I) PCNA-positive hepatocytes per field.
J Representative images showing Immuno-FISH using antibodies against γH2A.X and PCNA and Cy-3-labeled telomere-specific (CCCTAA) peptide nucleic acid probe.
K Representative image showing Immuno-FISH comparison between wild-type and $tlr2^{-/-}$ hepatocytes 72 h after CCl_4. Arrows indicate co-localization between γH2A.X and telomeres.
L–N Analysis of (L) γH2A.X, (M) mean number of TAF and (N) % of TAF-positive hepatocytes in mice under high-fat diet treated with neutrophil neutralizing antibody against Ly6G (or with IgG control).
O Representative p21 immunohistochemistry in mice under high-fat diet treated with neutrophil neutralizing antibody against Ly6G (or with IgG control). Scale bar = 100 µm.
P Quantification of p21-positive cells per field of view.

Data information: Data are mean ± SEM of $n = 3$–5 mice per group. Statistical analysis was performed using one-way ANOVA (Holm–Sidak method) for multiple comparisons and two-tailed t-test for single comparisons. $^{**}P < 0.01$; $^{*}P < 0.04$.

© 2021 The Authors

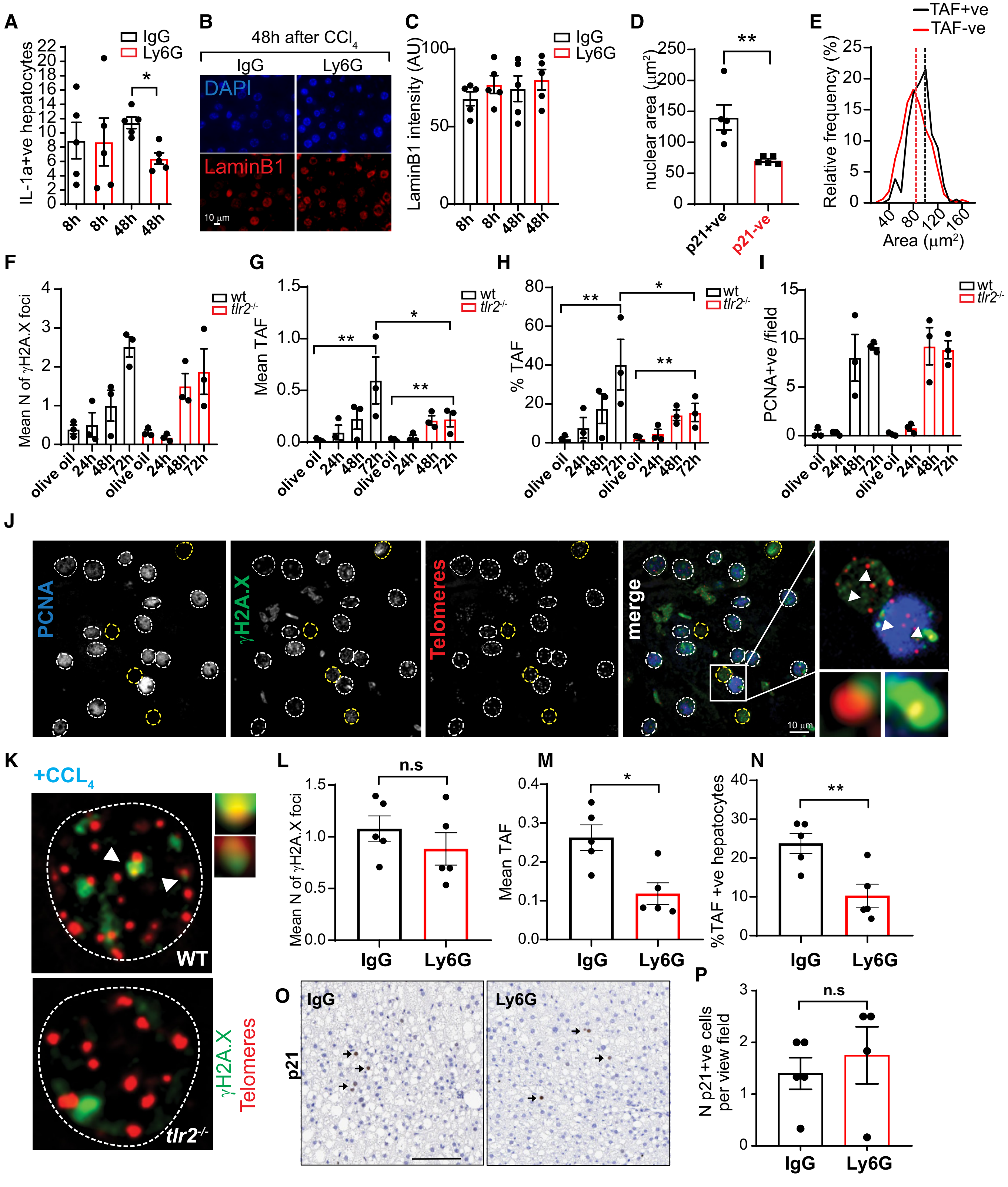
A
IgG
Ly6G
IL-1a+ve hepatocytes
8h
48h
B
48h after CCl4
IgG
Ly6G
DAPI
LaminB1
10 µm
C
LaminB1 intensity (AU)
D
nuclear area (µm²)
p21+ve
p21-ve
E
TAF+ve
TAF-ve
Relative frequency (%)
Area (µm²)
F
Mean N of γH2A.X foci
wt
tlr2-/-
olive oil
24h
48h
72h
G
Mean TAF
H
% TAF
I
PCNA+ve /field
J
PCNA
γH2A.X
Telomeres
merge
10 µm
K
+CCL4
WT
tlr2-/-
γH2A.X
Telomeres
L
n.s
Mean N of γH2A.X foci
M
Mean TAF
N
%TAF +ve hepatocytes
O
p21
P
N p21+ve cells per view field

Figure EV4.

Figure EV5. Supporting data for Figure 6.

A Quantification of mean number of TAF in hepatocytes as a function of the distance from a Ly6G labeled neutrophil. Data are from five mice aged 29 months of age, line indicates linear regression (linear regression and F test: $r^2 = 0.06$; $P = 0.0002$).

B Mean number of TAF (left) and mean number of γH2A.X foci (right) in aged (28–29 months old) *INK-ATTAC* mice treated with vehicle (VEH) or AP20187 (AP). Data are mean ± SEM of $n = 5$–6 mice per age group.

C Comparison between p21 mRNA levels of young (3 months old) and old INK-ATTAC mice (28–29 months old) treated with vehicle or AP20187. Data are mean ± SEM of $n = 5$–8 mice per age group.

D (left) Heat map demonstrating the distribution and relative intensity of the cell surface markers used in the clustering analysis in CyTOF experiment and (right) heat map showing the relative abundance of each cluster for each mouse (5 young and 5 old).

E Graph showing relative distribution of different intrahepatic leukocytes in young and old mice.

F (left) UMAP plots representing single-cell RNA-sequencing data obtained from (Ma *et al*, 2020) depicting different population in rat livers (5 and 27 months old). Neutrophils are labeled in red. (right) Violin plots show that $p16^{Ink4a}$ expression is very low in neutrophils at either age group.

G–J Pure LPS was administered by intraperitoneal injection to wild-type mice (8–10 weeks old; C57BL/6) at a dose of 300 μg/animal for 24 h: (G) Number of neutrophils infiltrates (NIMP+ve) in liver per field of view; (H) mean number of TAF and (I) % of TAF in hepatocytes 24 h following LPS injection; (J) representative images of Immuno-FISH using antibodies against γH2A.X and Cy-3-labeled telomere-specific (CCCTAA) peptide nucleic acid probe. Data are mean ± SEM of $n = 4$–5 mice per group.

Data information: Statistical analysis was performed using one-way ANOVA (Holm–Sidak method) for multiple comparisons and two-tailed *t*-test for single comparisons. $^{**}P < 0.01$; $^{*}P < 0.04$.

 © 2021 The Authors

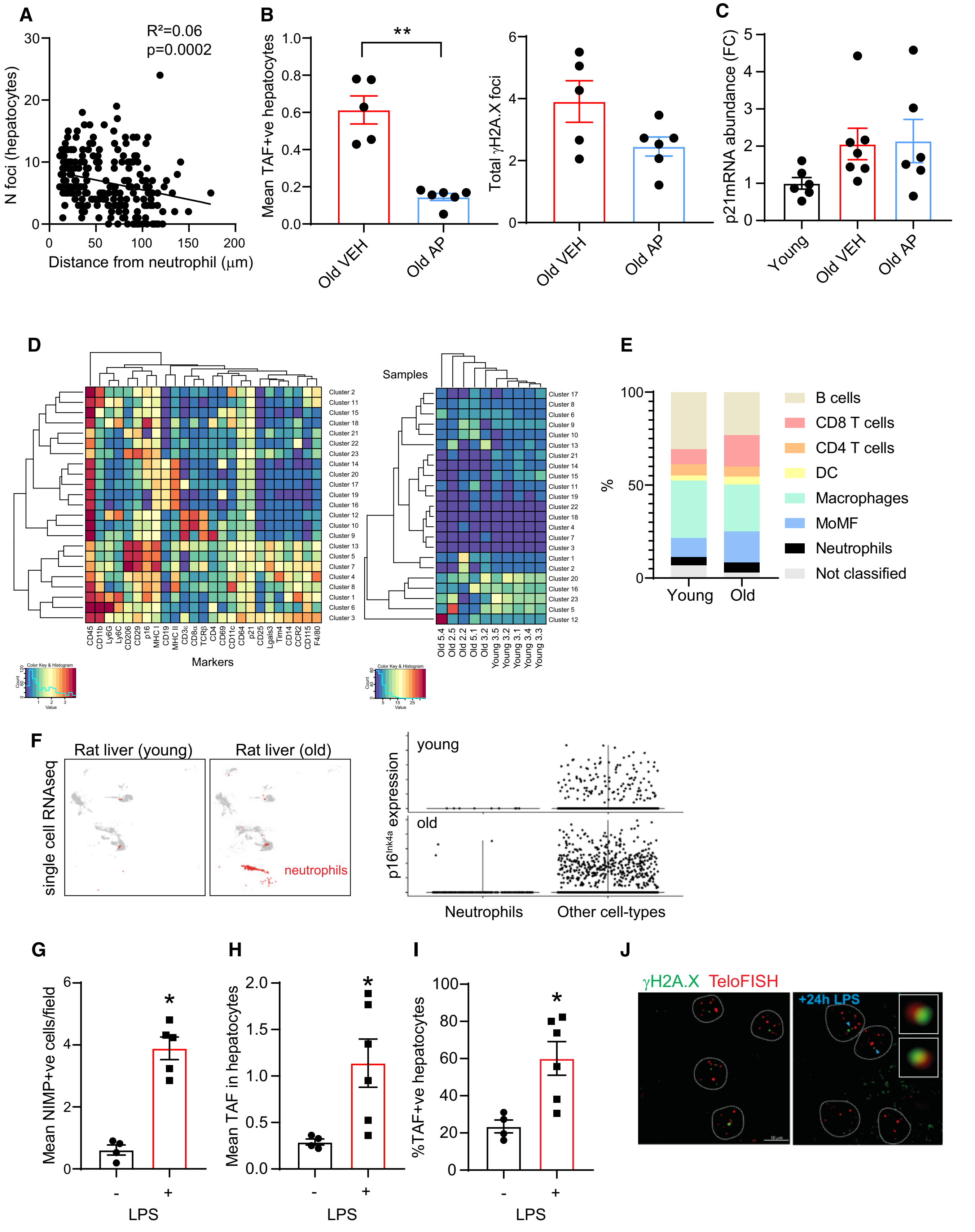
A
R²=0.06
p=0.0002
N foci (hepatocytes)
Distance from neutrophil (μm)
B
Mean TAF+ve hepatocytes
**
Total γH2A.X foci
Old VEH
Old AP
C
p21mRNA abundance (FC)
Young
Old VEH
Old AP
D
Samples
Markers
E
%
Young
Old
B cells
CD8 T cells
CD4 T cells
DC
Macrophages
MoMF
Neutrophils
Not classified
F
single cell RNAseq
Rat liver (young)
Rat liver (old)
neutrophils
young
old
p16Ink4a expression
Neutrophils
Other cell-types
G
Mean NIMP+ve cells/field
LPS
H
Mean TAF in hepatocytes
I
%TAF+ve hepatocytes
J
γH2A.X TeloFISH
+24h LPS

Figure EV5.
