# Supplementary figures and images for: Neutrophils induce paracrine telomere dysfunction and senescence in ROS‐dependent manner
Source: EMBO J. 2021 Mar 25;40(9):e106048. doi: 10.15252/embj.2020106048 (PMC8090854; doi:10.15252/embj.2020106048)

# uncropped western blots Fig1h

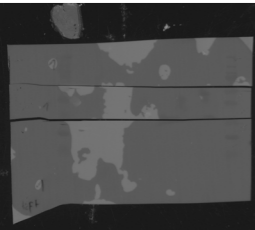

50

37

25

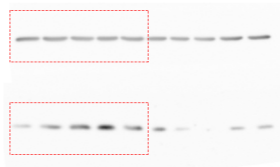

**GAPDH**

**p21**

Supplement: Supplementary file 4 — Source Data for Figure 1 [file EMBJ-40-e106048-s002.pdf]

# uncropped western blots Fig2m

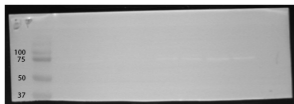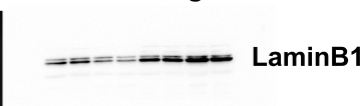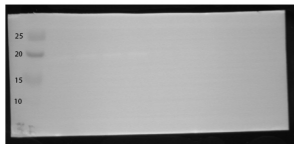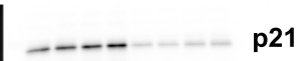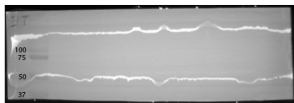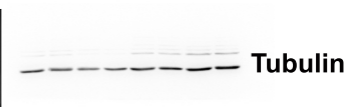

Supplement: Supplementary file 5 — Source Data for Figure 2 [file EMBJ-40-e106048-s005.pdf]
